# The role of Endobronchial ultrasound guided transbronchial needle aspiration (EBUS-TBNA) for qualitative diagnosis of mediastinal and hilar lymphadenopathy: a prospective analysis

**DOI:** 10.1186/1471-2407-11-100

**Published:** 2011-03-21

**Authors:** Ting Ye, Hong Hu, Xiaoyang Luo, Haiquan Chen

**Affiliations:** 1 Department of Thoracic Surgery, Fudan University, Shanghai Cancer Center; Shanghai Medical College, Fudan University, 270 Dong'an Rd, Shanghai, 200032, China; 2 Department of Oncology, Shanghai Medical College, Fudan University, 270 Dong'an Rd, Shanghai, 200032, China

## Abstract

**Background:**

Recently EBUS-TBNA, which has a sensitivity of 94.6%, specificity of 100% and diagnostic accuracy rate of 96.3% as previously reported, has been widely used for patients with mediastinal and hilar lymphadenopathy or suspected lung cancer to get accurate diagnosis. The purpose of the current study was to evaluate the usefulness of EBUS-TBNA in obtaining cytological and histological diagnosis of mediastinal and hilar lymph nodes compared to the results obtained with conventional mediastinoscopy as previously reported, and to assess the relationship of diagnostic accuracy and number of passes and size of lymph nodes.

**Methods:**

101 patients with mediastinal and hilar lymphadenopathy or suspected lung cancer in our institution were included in this prospective study. EBUS-TBNA was performed in all cases. The final diagnosis was confirmed by cytology, surgical results, and/or clinical follow-up for at least 6 months. Sensitivity, specificity, accuracy, and positive and negative predictive values were calculated using standard formulas.

**Results:**

In 101 patients, EBUS-TBNA was successfully performed to obtain samples from 225 lymph nodes, 7 lung masses, 1 mediastinal mass and 2 esophageal masses. 63 malignant tumors and 38 benign diseases were confirmed. Epidermal growth factor receptor mutation was detected in 10 biopsy samples, and epidermal growth factor receptor mutation was detected in 4 cases. With respect to the correct diagnosis of mediastinal and hilar lymphadenopathy, EBUS-TBNA had a sensitivity of 95.08%, specificity of 100%, positive predictive value of 100%, negative predictive value of 93.02%, and overall accuracy of 97.02%. The relationship of diagnostic accuracy and number of lymph node passes or size of lymph nodes was both insignificant (p = 0.27; p = 0.23). The procedure was uneventful without complications.

**Conclusions:**

EBUS-TBNA is an accurate and safe tool in diagnosis of mediastinal and hilar lymphadenopathy. It cannot completely replace mediastinoscopy, it may indeed reduce the number of mediastinoscopy procedures. In some cases, it can necessarily be the first-line procedure before mediastinoscopy.

## Background

Patients with mediastinal lymphadenopathy or suspected lung cancer required accurate diagnosis to determine optimal treatment. For these patients, mediastinal nodal sampling is often necessary and has traditionally been performed by mediastinoscopy or anterior mediastinotomy. However, mediastinoscopy, with a sensitivity of 80% to 85% and a specificity of nearly 100%, which is considered the gold standard for diagnosis with tissue confirmation of mediastinal lymphadenophy and lung cancer with mediastinal or hilar lymph nodes involved, does not allow access to all lymph node stations and is associated with a rate of morbidity that is not insignificant [1]. This situation has led to the promotion in recent years of minimally invasive techniques for mediastinal lymph node evaluation.

Real-time endobronchial ultrasound guided transbronchial needle aspiration (EBUS-TBNA) is a new technique that combines endoscopic visualization with high frequency ultrasound imaging, which is used to obtain cytological and histological samples of lesions adjacent to the tracheobronchial tree [2-4]. This makes it easier to locate the lymph nodes to be sampled. As Yasufuku and colleagues [4] reported, EBUS-TBNA had a sensitivity of 94.6%, specificity of 100% and diagnostic accuracy rate of 96.3%, which seemed to be superior to those of mediastinoscopy. However, whether EBUS-TBNA can be applied as the first-line procedure for diagnosis of mediastinal lymphadenopathy is still controversial, because of its false negative rate to some extent [5]. Moreover, there is few studies reported comparing the relationship of diagnostic accuracy and number of passes or size of lymph nodes. The main aim of our study was to evaluate the role of EBUS-TBNA in obtaining cytological and histological diagnosis of mediastinal lymph nodes compared to the results obtained with conventional mediastinoscopy as previously reported, and to assess the relationship of diagnostic accuracy with number of passes and size of lymph nodes.

## Methods

### Patients

Patients having mediastinal lymphadenopathy or with mediastinal or hilar lesion suspected of lung cancer detected on enhanced thoracic CT were included in this prospective study. Between March to October 2009, 101 patients in our institution met this inclusion criteria and were included in this study. The study was approved by the ethical committee of Fudan University Shanghai Cancer Center and a written informed consent was obtained in all the patients.

### EBUS-TBNA procedure

EBUS-TBNA was performed under venous anaesthesia. Patients were monitored for electrocardiogram, pulse oximetry, and blood pressure with the presence of an anesthesiologist. A flexible bronchoscope containing an ultrasound probe (XBF-UC206F-OL8; Olympus) was inserted via the laryngeal mask and guided through the trachea and the bronchial tree towards the appropriate area of the mediastinum. The targeted lymph nodes or masses were identified using bronchoscopic visualization and ultrasound imaging. A needle (NA-201SX-4022; Olympus) extended from the bronchoscope through the bronchial wall was used to puncture the lesion and to aspirate tissue. A lymph node or mass could be punctured three to four times to gain an adequate sample, and several lymph nodes could be punctured during the same session. The aspirates were then smeared on slides and simultaneously sent to pathology laboratory for subsequent cytology. The cytology sample was considered adequate if it contained malignant cell or a large number of lymphoid cells confirmed by the cytologist, and then the operation is terminated. The lymph nodes punctured were grouped according to the puncture site: the upper paratracheal (2R, 2L), the subcarinal station (7), the lower paratracheal and hilar station (4R, 4L, 10R, and 10L), the right paraesophageal (8R) and the interlobar station (11L,11R).

Histological cores were collected and fixed with formalin and stained with hematoxylin and eosin for further histology examination. In some cases, immunohistochemistry was performed for additional information, and EGFR mutation was also evaluated.

### Statistical analysis

The EBUS-TBNA diagnosis was confirmed by cytology, surgical results, and/or clinical follow-up for at least 6 months. A positive cytological result of malignancy was accepted as evidence, and the patients were treated accordingly.

The data were entered into a database and analyzed with the SPSS statistical software package (SPSS 15.0 Chicago, Illinois, USA). A descriptive analysis was carried out in which categorical variables were expressed as absolute and relative frequencies, and continuous variables as means (SD). The χ2 was used to compare proportions in independent groups. The spearman analysis was used to compare correlation between two independent groups. A P value of less than 0.05 was considered significant. Sensitivity, specificity, accuracy, and positive and negative predictive values were calculated using standard formulas.

## Results

### Patient characteristics

We studied consecutively all the patients who underwent EBUS-TBNA for the evaluation of mediastinal and/or hilar lymph nodes and mediastinal/hilar lesion on an inpatient basis between March to October 2009. A total of 101 patients with a mean age of 57.4 years (range, 24-84) were enrolled in this study, including 68 males and 33 females. Clinically, among these patients, 55 patients were suspected for lung cancer, 2 patients were suspected for esophageal carcinoma, one patient suffered from a malignant mediastinal mass and 43 benign mediastinal lymphadenopathy, who needed pathological diagnosis (see Table 1). All patients were followed up for at least 6 months.

**Table 1:** Patient characteristics and pre-operation diagnosis

Patient characteristics	
Patients	N = 101
Male/female gender	68/33
Median age(year)(range)	57.4 years (24-84)

**Pre-operation diagnosis**	

Suspected for lung cancer	N = 55
Suspected for esophageal carcinoma	N = 2
Malignant mediastinal mass	N = 1
Mediastinal and hilar lymphadenopathy	N = 43

Pre-operation diagnosis: mainly based on CT feature

### Operation parameters

A total of 225 lymph nodes, 7 lung masses, 1 mediastinal mass and 2 esophageal masses were biopsied. Details of lymph node stations and masses punctured are shown in Tables 2 &3(see Table 2 &3). 151 lymph nodes were punctured once, and 74 lymph nodes were punctured more than once (range, 2-5) (see Table 4). Diameter of lymph nodes ranged from 0.6 cm to 10.5 cm with a median diameter of 2.04 cm. There were 154 lymph nodes of which maximal diameter was <2 cm, and 71 lymph nodes of which maximal diameter was >2 cm (see Table 5). Mean period of each TBNA passes was 4.9 minutes and mean stay length in hospital was 2 days. Mediastinoscopy was conducted in 23 patients, video-assisted thoracotomy surgery (VATS) was conducted in 2 patients and 2 patients received transbronchial lung biopsy (TBLB) procedure. No procedure-related complications such as pneumothorax, pneumomediastinum or excessive bleeding ever occurred in this study (see Table 2 &3).

**Table 2:** Operation parameters, further confirmation modalities, lymph node size and diagnostic yield

Operation parameters	
Number of lymph nodes biopsied	N = 225
Number of lung mass biopsied	N = 7
Number of esophageal masses biopsied	N = 2
Number of mediastinal masses biopsied	N = 1
Mean period of each TBNA pass (min)	4.9
Mean stay length in hospital (day)	2.0
Complications	No case

**Further confirmation modalities**	

Mediastinoscopy	N = 23
VATS	N = 2
TBLB	N = 2
6-months follow-up	N = 74

**Lymph node size (mm)**	

Long axis	20.4 (6-105)
Short axis	17 (6-50)

**Diagnostic yield %**	

Sensitivity	95.08
Specificity	100
Accuracy	97.02
Positive predictive value (PPV)	100
Negative predictive value (NPV)	93.02

VATS: video-assisted thoracotomy surgery; TBLB: transbronchial lung biopsy

**Table 3:** Location of Lymph Node Station Biopsied by EBUS-TBNA and number of TBNA passes of each Lymph Node Station

Right upper paratracheal (2R)	N = 42	Left upper paratracheal (2L)	N = 1
Right lower paratracheal (4R)	N = 110	Left lower paratracheal (4L)	N = 41
Subcarina (7)	N = 80	Right paraesophageal (8R)	N = 2
Right hilar (10R)	N = 16	Left hilar (10L)	N = 7
Right interlobar (11R)	N = 4	Left intherlobar (11L)	N = 2
		Total	N = 305

EBUS-TBNA: endobronchial ultrasound guided transbronchial needle aspiration

**Table 4:** The relationship of diagnostic accuracy and number of lymph node passes

	Number of lymph node passes	
	N = 1	N > 1
Total (N = 225)	151	74
Successful passes	117	61
Unsuccessful passes	34	13
Diagnostic accuracy	77.5%	82.4%

P = 0.27		

N > 1 including N = 2-5

**Table 5:** The relationship of diagnostic accuracy and size of lymph nodes

	Maximal diameter of lymph nodes	
	≦2 cm	>2 cm
Total (N = 225)	154	71
Successful passes	119	60
Unsuccessful passes	35	11
Diagnostic accuracy	77.3%	84.5%
P = 0.23		

The spearman correlation analysis was used to compare correlation between the two groups

### Diagnostic yield

According to the cytological and histological results, 63 malignant tumors and 38 benign diseases were confirmed. Of these 63 patients, there were 24 cases of adenocarcinoma, 5 cases of squamous carcinoma, 20 cases of small cell lung cancers, 9 cases of undifferentiated carcinoma, one case of renal carcinoma with mediastinal lymph node metastasis and two cases of lymphoma. There were 38 patients with benign diseases: 13 cases with no malignancy evidences, 14 cases with granulomatous inflammation, 4 cases with sarcoidosis, 4 cases with tuberculosis, one small B cell tumor, one lymph node hyperplasia and one chronic lymph node inflammation. Meanwhile, EBUS-TBNA failed to reveal one small cell lung cancer, one squamous carcinoma, one undifferentiated carcinoma, which were finally diagnosed by further confirmatory tests (see Table 6).

**Table 6:** Final cytological and histological results

Malignancy (N = 61)	
Adenocarcinoma	N = 24
Small cell lung cancer	N = 20
Squamous carcinoma	N = 5
Undifferentiated carcinoma	N = 9
Renal carcinoma metastasis	N = 1

Benign diseases (N = 40)	

No MT evidence	N = 13
Granulomatous inflammation	N = 14
Sarcoidosis	N = 4
Tuberculosis	N = 4
Lymphoma	N = 2
Small B cell tumor	N = 1
Lymph node hyperplasia	N = 1
Chronic inflammation	N = 1

The sensitivity of real-time EBUS-TBNA was 95.08% and specificity was 100%. Also, the positive predictive value and the negative predictive value was 100% and 93.02%, respectively. The overall accuracy was 97.02% (see Table 2). The relationships of diagnostic accuracy with number of lymph node passes and with size of lymph nodes were both insignificant (p = 0.27; p = 0.23) (see Table 4&5).

### EGFR mutation measurement

EGFR mutation was detected in 10 EBUS-TBNA biopsy samples which were confirmed adenocarcinoma on cytological level. In 4 cases, EGFR mutation (40%) was confirmed. One was heterozygosity deletion (E752-E759del) of exon 19, one was heterozygosity deletion (E746-E750del) of exon 19, and two were point mutations (L858R) of exon 21. No EGFR mutation was found in the other six patients (see Table 7).

**Table 7:** Results of EGFR mutation detection on the cytological level

EGFR mutation status	Number
Heterozygosity Deletion (E752-E759del) of Exon 19	1
Heterozygosity Deletion (E746-E750del) of Exon 19	1
Point Mutations (L858R) of Exon 21	2
Negative	6

## Discussion

For many years surgical biopsy - principally mediastinoscopy - has been regarded as the "standard procedure" for sampling mediastinal lymph nodes [1]. However, mediastinoscopy can only sample nodal stations 1-4, 7, access to hilar nodal stations could be difficult and may require thoracoscopy and on occasion a thoracotomy. Moreover, it cannot be repeatedly operated on the same patient [5,6]. Contrarily, EBUS-TBNA, when combined with EUS, can sample all the key nodal stations and also can be performed repeatedly [7,8]. In our study, 225 lymph nodes were biopsied and 10 stations of mediastinal and hilar nodes were punctured (see Table 2 &3). Moreover, nearly every lymph nodal group had been checked. And almost every patient was biopsied three to four lymph node stations. A total number of 305 TBNA passes of lymph nodes were conducted (see Table 3). On the other hand, mediastinoscopy is more invasive than endoscopic techniques and results in a neck scar which may be cosmetically unacceptable to some patients. Unfortunately it does have a 2% risk of morbidity and 0.08% mortality [1,9,10]. In this study, 101 patients received EBUS-TBNA, of which mediastinoscopy was conducted in 23 patients, 2 patients received VATS and 2 patients received TBLB procedure, no procedure-related complications ever occurred (see Table 2). The mean period of each EBUS-TBNA was 4.9 minutes and mean stay length in hospital was 2 days, which seemed to be more minimal invasive, compared with those parameters of mediastinoscopy.

Furthermore, though viewed as the gold standard for mediastinal nodal assessment, the diagnostic sensitivity of cervical mediastinoscopy is only 78-81%, which is inferior to that of EBUS-TBNA, as reported in two recent systematic reviews [1,11]. Mediastinoscopy has a negative predictive value of 91% which is also inferior to that of endoscopic techniques thus far [1,11]. Similarly found in previous studies [12,13], the sensitivity of real-time EBUS-TBNA in our study was 95.08% and the negative predictive value was 93.02% (see Table 8). This diagnostic yield was obviously in favor of EBUS-TBNA over cervical mediastinoscopy. However, current data was not consistent. Another study of 33 patients evaluating EBUS versus cervical mediastinoscopy for nodal staging of patients with suspected or confirmed lung cancer revealed a lower sensitivity and slightly inferior negative predictive value for EBUS with similar accuracies (90.9 versus 93.9% for EBUS and cervical mediastinoscopy, respectively). In the study, the prevalence of N2 or N3 disease was lower at 39.4%. Importantly, three patients were upstaged by cervical mediastinoscopy from N0 (on EBUS) to N2, indicating it may not completely replace cervical mediastinoscopy [14]. According to our study, 46 patients with suspected or confirmed lung cancer received EBUS-TBNA, 2 patients with confirmed lung cancer for restaging after chemotherapy, and confirmatory EBUS-TBNA was operated on 43 patients with preoperatively suspected mediastinal diseases. Besides, 7 lung masses, 1 mediastinal mass and 2 esophageal masses were biopsied by EBUS-TBNA. Regarding the final cytological and histological results showed above, EBUS-TBNA successfully diagnosed 58 malignant tumors and failed to reveal one small cell lung cancer, one squamous carcinoma, one undifferentiated carcinoma, which were finally diagnosed by further confirmatory tests (see Table 9). The malignancy prevalence was 60.4% in our study. Compared with those different results, in our study, it was suggested that the variability in performance may relate to the significant variation in disease prevalence. When disease prevalence was high, existing data favored EBUS but when it was moderate then cervical mediastinoscopy appeared superior. (See Table 10)

**Table 8:** Diagnostic Ability of Endobronchial Ultrasound Guided Transbronchial Needle Aspiration: Comparison With Previous Studies

%							
**Author**	**Year**	**N**	**Sensitivity**	**Specificity**	**PPV**	**NPV**	**Accuracy**

Yasufuku [4]	2005	108	94.6	100	100	89.5	96.3
Vincent [12]	2008	152	95	100	100	97	98.7
Andrade [13]	2008	46	96.4	100	100	92.5	97.8
Our study	2009	101	95.08	100	100	93.02	97.02

PPV: positive predictive value; NPV: negative predictive value

**Table 9:** Comparison of real-time EBUS-guided TBNA results with final diagnosis of all patients

	EBUS-TBNA results		
Final diagnosis	malignant	benign	Total
Malignant	58	3	61
Benign	0	40	40
Total	58	43	101

**Table 10:** Comparative diagnostic performance of EBUS-TBNA and mediastinoscopy

Technique	Nodes	Risks	Sensitivity	NPV	Prevalence
Mediastinoscopy [1]	1-4, 7	0.08% mortality and 2% morbidity: arrhythmias [15], haemorrhage, thoracic duct injury, recurrent laryngeal nerve injury, pneumonia, bronchial/pleural laceration [1]	78-81%	91%	39%
EBUS-TBNA	2-4, 7, 10-12	None	95.08%	93.02%	60.4%

EBUS-TBNA: based on data from our study; Mediastinoscopy: based on data from previous studies

Another unsettled point is that whether EBUS-TBNA can replace mediastinoscopy as first-line procedure for diagnosis benign mediastinal diseases such as sarcoidosis, tuberculosis, etc. As Nakajima and colleagues reported in 2009 [15], EBUS-TBNA should be added to conventional diagnostic modalities for patients with suspicious stage I sarcoidosis on chest roentgenogram. In their study, they compared EBUS-TBNA with TBLB and BAL and got their conclusion, but they did not compare with mediastinoscopy. In our research, 38 benign diseases were diagnosed. And 5 cases of granulomous inflammation were diagnosed by EBUS-TBNA. Also, of 4 patients with tuberculosis, one case was diagnosis by EBUS-TBNA. Since non-caseating granuloma can be detected by EBUS-TBNA, sarcoidosis and tuberculosis can be diagnosed with the combination of clinical features and laboratory evidences.

Since there are few studies analyzing the relationship between diagnostic accuracy and number of lymph node passes or size of lymph nodes reported previously, we assessed the correlation of diagnostic accuracy and the two parameters above. Though the diagnostic accuracy of EBUS-TBNA should be higher when the lymph node size was larger or number of passes was more on the theoretical basis, we found that the relationship of diagnostic accuracy and number of passes was insignificant (see Table 4 &5). Similarly, there was no significant correlation between diagnostic accuracy and size of lymph nodes. Our results suggest that neither number of lymph node passes nor size of lymph nodes be the key factor which determines the success of EBUS-TBNA. The certainty that the needle is punctured into the lymph node and enough samples are collected during EBUS-TBNA procedure is much more important.

Another potential advantage of EBUS-TBNA is that the cytological samples can provide molecular biological information that will possibly be useful for the treatment of advanced lung cancer. In our study, we detected EGFR mutation in ten biopsy samples. We found 4 EFGR mutation cases: one heterozygosity deletion (E752-E759del) of exon 19, one heterozygosity deletion (E746-E750del), and two point mutations (L858R) of exon 21. We detected EGFR mutation on the cytological level, which was different from what Nakajima reported on the histological level in 2007 [16]. Though whether EGFR mutation found in metastatic lymph nodes is accordance with that of the primary tumor is still unknown, however, molecular biological information provided by EBUS-TBNA is evidently of great value and maybe it can guide targeted therapy for advanced lung cancer patients. Obviously more researches are needed for further confirmation.

## Conclusions

EBUS-TBNA was an accurate and safe tool in diagnosis of mediastinal lymphadenopathy and lung cancer. Surely EBUS-TBNA cannot completely replace mediastinoscopy so far, it may indeed reduce the number of mediastinoscopy procedures. In patients with positive lymph nodes suspected by enhanced thoracic CT and PET/CT, it can necessarily be the first-line procedure before mediastinoscopy.

## Competing interests

The authors declare that they have no competing interests.

## Authors' contributions

All authors have read and approved the final manuscript.

TY: designation and progress of the study; data collection; patients' follow-up and writing

HH: designation and progress of the study and revision of this manuscript

XYL: data collection and patients' follow-up

HQC: designation and progress of the study; evaluation of this study; assessment and revision of the manuscript

## Pre-publication history

The pre-publication history for this paper can be accessed here:

http://www.biomedcentral.com/1471-2407/11/100/prepub

## References

[B1] Detterbeck FC Jantz MA Wallace M American College of Chest Physicians. Invasive mediastinal staging of lung cancer: ACCP evidence-based clinical practice guidelines Chest 2007 132 2 3 Suppl 202S 220S 10.1378/chest.07-1362 17873169

[B2] Yasufuku K Chiyo M Sekine Y Real-time endobronchial ultrasound-guided transbronchial needle aspiration of mediastinal and hilar lymph nodes Chest 2004 126 122 128 10.1378/chest.126.1.122 15249452

[B3] Rintoul RC Skwarski KM Murchison JT Endobronchial and endoscopic ultrasound-guided real-time fine-needle aspiration for mediastinal staging Eur Respir J 2005 25 416 421 10.1183/09031936.05.00095404 15738283

[B4] Yasufuku K Chiyo M Koh E Endobronchial ultrasound guided transbronchial needle aspiration for staging of lung cancer Lung Cancer 2005 50 347 354 10.1016/j.lungcan.2005.07.013 16171897

[B5] Medford A Bennett JA Free CM Mediastinal staging procedures in lung cancer: EBUS, TBNA and mediastinoscopy Curr Opin Pulm Med 2009 15 334 342 10.1097/MCP.0b013e32832b8a45 19395972

[B6] Ernst A Anantham D Eberhardt R Diagnosis of Mediastinal Adenopathy--Real-time endobronchial ultrasound guided needle aspiration versus mediastinoscopy J Thorac Oncol 2008 3 577 582 10.1097/JTO.0b013e3181753b5e 18520794

[B7] Herth FJ Lunn W Eberhardt R Transbronchial versus transesophageal ultrasound-guided aspiration of enlarged mediastinal lymph nodes Am J Respir Crit Care Med 2005 171 1164 1167 10.1164/rccm.200411-1560OC 15665318

[B8] Yasufuku K Fujisawa T Staging and diagnosis of non-small cell lung cancer: invasive modalities Respirology 2007 12 173 183 10.1111/j.1440-1843.2007.01035.x 17298448

[B9] Hammoud ZT Anderson RC Meyers BF The current role of mediastinoscopy in the evaluation of thoracic disease J Thorac Cardio-vasc Surg 1999 118 894 899 10.1016/S0022-5223(99)70059-0 10534695

[B10] Porte H Roumilhac D Eraldi L The role of mediastinoscopy in the diagnosis of mediastinal lymphadenopathy Eur J Cardiothorac Surg 1998 13 196 199 10.1016/S1010-7940(97)00324-2 9583827

[B11] Toloza EM Harpole L Detterbeck F Invasive staging of nonsmall cell lung cancer: a review of the current evidence Chest 2003 123 1 Suppl 157S 166S 10.1378/chest.123.1_suppl.157S 12527575

[B12] Vincent BD El-Bayoumi E Hoffman B Real-time endobronchial ultrasound-guided transbronchial lymph node aspiration Ann Thorac Surg 2008 85 224 230 10.1016/j.athoracsur.2007.07.023 18154815

[B13] Groth SS Whitson BA D'Cunha J Endobronchial ultrasound-guided fine-needle aspiration of mediastinal lymph nodes: a single institution's learning curve Ann Thorac Surg 2008 86 1104 1109 10.1016/j.athoracsur.2008.06.042 18805141

[B14] Yasufuku K Quadri M dePerrot M A prospective controlled trial of endobronchial ultrasound guided transbronchial needle aspiration compare to mediastinoscopy for mediastinal lymph node staging of lung cancer Western Thoracic Surgical Association 33rd Annual Meeting (abstract) Canada 2007

[B15] Nakajima T Yasufuku K Kurosu K The role of EBUS-TBNA for the diagnosis of sarcoidosis - comparisons with other bronchoscopic diagnostic modalities Respiratory Medicine 2009 103 1796 1800 10.1016/j.rmed.2009.07.013 19674882

[B16] Nakajima T Yasufuku K Suzuki M Assessment of epidermal growth factor receptor mutation by endobronchial ultrasound-guided transbronchial needle aspiration Chest 2007 132 597 602 10.1378/chest.07-0095 17573511

